# Deep learning for steganalysis: evaluating model robustness against image transformations

**DOI:** 10.3389/frai.2025.1532895

**Published:** 2025-03-26

**Authors:** Othman A. Alrusaini

**Affiliations:** Department of Engineering and Applied Sciences, Applied College, Umm Al-Qura University, Makkah, Saudi Arabia

**Keywords:** steganalysis, deep learning, image transformations, model robustness, digital security, EfficientNet

## Abstract

This study investigates the robustness of deep learning-based steganalysis models against common image transformations because most literature has not paid enough attention to resilience assessment. Current and future applications of steganalysis to guarantee digital security are gaining importance regarding real-world modifications: resizing, compression, cropping, and adding noise. These included the following five basic models: EfficientNet, SRNet, ResNet, Xu-Net, and Yedroudj-Net. We evaluated these models' pre- and post-transformation performances based on various metrics like accuracy, precision, recall, F1-score, and AUC with the BOSSBase dataset. Our results showed that EfficientNet is the most robust among the considered architecture transformations. Still, it also underlined significant degradations for state-of-the-art models, Xu-Net and Yedroudj-Net, especially with added noise. These results indicate the need to develop more robust architectures capable of sustaining real-world image alterations. In practice, it will assist practitioners in choosing models that best suit operational environments and lay the necessary platform for future enhancements in the design of such models. In this regard, in the future, more transformations should be researched with ensemble and adaptive approaches to improve robustness further.

## 1 Introduction

The word steganography comes from the Greek word for “covered writing.” This methodology intends to hide messages within other non-suspicious media as images to mask their very existence. While cryptography works to keep the content of a message safe, steganography keeps the message undetectable so as not to be intercepted in the first place (Wu et al., 2018). Most modern steganographic approaches are incomparably more complicated, either by embedding data in the least significant bits of image pixels or by more sophisticated transformations that ensure the undetectability of messages. While steganography techniques are evolving, steganalysis models are also evolving by leveraging machine learning and deep learning for accurate detection (Alzubi et al., 2023). However, most of these models still face the challenge of resilience regarding practical conditions in digital security (De La Croix et al., 2024; Zou et al., 2019).

In practice, images are usually exposed to typical transformations, such as rescaling, compression, cropping, and adding noise, before being analyzed for steganographic content. These may include resizing, which distorts the coherency of hidden data in space and hence masks the detection. It is, however, still possible that compression, especially in lossy formats such as JPEG, might strip out the embedded information by removing subtle variations in pixels carrying the messages (Zeng et al., 2017). Removal of image segments by cropping may omit regions where data are stored while adding noise mask signals through random changes in pixel values that further complicate detection processes. These transformations daunt these steganalysis models, which normally would have been trained on unaltered images, resulting in low accuracy upon the occurrence of any transformed media (Li et al., 2024).

This work investigates the challenging gap in steganalysis—the robustness of the steganalysis model against common image transformations. The following are the key research questions considered for investigation in this study:

1. Impact of Transformations: How do different transformations of images, such as resizing, compression, cropping, and noise addition, affect the accuracy and robustness of steganalysis models?
2. Model Robustness: Which models are more robust against different transformations while maintaining detection accuracy?
3. Identification of Transformation-Resilient Models: Is it possible to pinpoint any models or architecture of models that are more resilient against image transformations, and what characteristics give them resilience?
4. Guidelines for Practical Steganalysis: What best practices can be derived to guide the application of steganalysis in dynamic real-world environments where images are most likely to transform?

These questions target filling the gap between artificially controlled experimental conditions and real-world applications in furthering the area of steganalysis by finding transformation-robust models.

The paper is organized as follows: in Section 2, we include a review of the related literature on steganography, steganalysis, and the impact of transformations; Section 3 includes a description of methodology, data, and models; Section 4 includes results; Section 5 includes a discussion of implications. In the conclusion section, we summarize our findings and indicate some promising avenues for future research.

## 2 Literature review

### 2.1 Overview of steganography and steganalysis

Steganography moved beyond the conventional Least Significant Bit (LSB) algorithm that inserted hidden data in pixel value bits to current adaptive techniques that modify image structures for better cloaking purposes (Hu et al., 2019; Chaumont, 2020). The initial steganographic approach using LSB depended on basic pixel modification. However, forensic analysis evolution brought Highly Undetectable Steganography (HUGO) and Universal Wavelet Relative Distortion (UNIWARD) as adaptive embedding systems that prevent statistical indicators of message concealment (Dhawan et al., 2021). The evolution of steganalysis methods required new developments because it shifted away from traditional statistical detection toward machine learning tools. The detection ability of Regular and Singular (RS) statistical methods proved insufficient to identify modern adaptive steganographic techniques compared to the results achieved (Chen et al., 2023). Support vector machines (SVMs) alongside ensemble classifiers represented machine learning (ML) detection methods, which improved the process yet depended on manual feature extraction. Deep learning methods through convolutional neural networks (CNNs) enable automatic feature extraction, leading to proven steganographic pattern detection in images without requiring predefined statistical metrics (Pibre et al., 2020; Zou et al., 2019; Alzubi, 2022).

### 2.2 Deep learning in steganalysis

Deep learning models have advanced steganalysis detection by performing automated feature extraction while eliminating limited statistical patterns (Pibre et al., 2020). The pixel intensity variation analysis used by Regular and Singular (RS) steganalysis could not adapt to contemporary steganographic methods and provided inadequate detection performance (Chen et al., 2023). CNNs became instrumental in steganalysis transformation because they learn spatial frequency features directly from photographs without requiring prebuilt features. Xu-Net represents an early CNN-based steganalysis model that analyzes high-frequency artifacts, yet it shows limitations when working with transformed images, thus affecting its generalization capability (Wani and Sultan, 2023). The pre-processing layers of Yedroudj-Net boost its feature learning abilities, although the system suffers from compression attacks and resizing issues (Vijjapu et al., 2023). SRNet and ResNet represent advanced architectural models that improve resistance in steganographic detection because SRNet performs exceptional multi-scale spatial analyses (Hu et al., 2018) and ResNet maintains deep feature representations through residual connections (Kuchumova et al., 2024). The compound scaling method implemented in EfficientNet enables better generalization during transformations because it optimizes depth and resolution parameters (Selvaraj et al., 2021).

### 2.3 Feature extraction mechanisms in steganalysis models

Deep learning-based steganalysis models operate through effective feature extraction because it determines their detection capabilities toward steganographic content and their resistance against transformations of images (Alzubi et al., 2022). Steganalysis models using hierarchical feature extraction methods obtain enhanced robustness against real-world image distortions because they detect low-scale pixel changes combined with high-scale structural analytics (Saxena et al., 2023). The deep hierarchical extraction used in EfficientNet and SRNet models creates a strong resistance against common image distortions, including resizing, compression, and cropping. The compound scaling method of EfficientNet achieves optimal depth, width, and resolution adjustments to maintain pixel dependencies and spatial relationships throughout the model process (Selvaraj et al., 2021). The high-frequency artifact detection approach used by Xu-Net and Yedroudj-Net makes them sensitive to compression and noise addition because these disturbances damage essential patterns necessary for accurate steganalysis (Tabares-Soto et al., 2021). Allocation functions of Swish in EfficientNet and SRNet help preserve information when gradients flow smoothly. In contrast, the ReLU activation in Xu-Net and Yedroudj-Net leads to performance degradation from gradient vanishing (Huang et al., 2024). The squeeze-and-excitation (SE) layers embedded in EfficientNet enable dynamic feature importance calibration, making the model better adapt to critical steganographic signals even after compression or noise addition transformations (Selvaraj et al., 2021). The robustness of EfficientNet and SRNet exceeds that of Xu-Net and Yedroudj-Net since their restricted architectural complexity fails to match the models' ability to preserve detection accuracy under spatial inconsistencies.

### 2.4 Impact of image transformations on steganalysis models

Multiple image transformations, including resizing, compression, cropping, and noise addition negatively impact steganalysis models due to their ability to change pixel statistics and break steganographic code sequences. The spatial structure of pixels gets disrupted after resizing because this process distorts important embedding artifacts, thus leading to decreased detection accuracy (Dengpan et al., 2019). Dealing subliminal data in frequency components occurs through JPEG's lossy compression because the process removes unneeded image data (Zeng et al., 2017). When cropping an image, detection models become misdirected because the process removes potentially hidden information within the deleted sections (Liu et al., 2021). Steganographic data becomes imperceptible when noise is added to images because such variations make it difficult for detection models with spatial consistency to identify hidden content (Tabares-Soto et al., 2021). EfficientNet shows enhanced robustness because its compound scaling and squeeze-and-excitation layers ensure accurate adaptive feature recalibration (Selvaraj et al., 2021). The Xu-Net and Yedroudj-Net demonstrate maximum vulnerability when facing transformations of their minimal hierarchical structure and dependence on high-frequency components (Tabares-Soto et al., 2021). The residual connections inside ResNet and SRNet protect essential features from damage, yet noise contamination remains a significant problem for all examined models (Kuchumova et al., 2024).

### 2.5 Adversarial machine learning in steganalysis

Deep learning-based steganalysis encounters an increasing difficulty from adversarial machine learning because attackers can modify images, which causes detection models to become misdirected. Adversarial attacks use tiny, unperceivable modifications on images, making deep learning models falsely identify stego and cover images and significantly reduce detection success (De La Croix et al., 2024; Qin et al., 2020; Alzubi et al., 2024). Attackers use technological vulnerabilities in CNN-based architecture to subtly modify learned feature patterns that result in detection avoidance. The main defense against adversarial attacks uses adversarial training to expose deep learning systems to clean and artificially perturbed images, enhancing their ability to resist such threats (Eid et al., 2022). Through this method, the model gains improved capabilities to detect modified data while enhancing its functionality in challenging attack situations. GANs are a mechanism to develop adversarial perturbations that mimic steganographic elements that enhance data variety and model resistance (Kuchumova et al., 2024). One can detect steganographic content using advanced adaptive methods by employing GAN-based steganalysis. The stronger robustness achieved through adversarial learning comes with increased difficulty during computation and an ongoing need for adapting to new enemy approaches.

### 2.6 Machine learning feature selection in steganalysis

The success of steganalysis models depends heavily on feature selection because it boosts detection precision through improved computation performance. Models using semi-supervised feature selection methods produce better outcomes while using unannotated data in combination with labeled data (Farooq and Selwal, 2023). Such techniques extract data's most useful stego detection features by eliminating excessive or noisy elements to improve model predictive abilities. The hypergraph Laplacian-based feature discrimination technology enables superior feature relationship detection using pixel value mathematics to depict high-order dependencies (Mikhail et al., 2023). The improved feature contrast in this method assists steganalysis models in detecting stego images from cover images more efficiently when applied to different transformations. Model efficiency is boosted through dimensionality reduction techniques such as Principal Component Analysis (PCA) and autoencoders because these methods select only relevant features to eliminate unimportant ones (Li et al., 2024). These methods diminish computational charges and boost model stability by overfitting limitations, especially for deep learning-based steganalysis applications. Choosing optimal features across multiple datasets presents challenges for researchers who need to explore better automatic and data-driven feature selection methods.

### 2.7 Research gaps

The current state of deep learning-based steganalysis models faces crucial obstacles while handling genuine image transformations because they generally operate on untampered datasets yet encounter difficulties when presented with compression, resizing, and noise impairments (Farooq and Selwal, 2023). Detected performance remains weak because adversarial attacks and lack of adversity robustness combine to create small imperceptible perturbations that lead steganalysis models into misclassifying content (De La Croix et al., 2024). Domain adaptation techniques need to be implemented because they strengthen networks through training with multiple image transformations, improving model performance in different operational conditions. Self-supervised learning has proven to be a promising solution because it enables models to extract robust data representations from unlabeled information sources while omitting the need for extensive manual labeling of datasets (Mikhail et al., 2023). Ensemble learning techniques utilizing several steganalysis models enhance generalization by combining different detection methods (Eid et al., 2022). The development of steganalysis solutions should prioritize integrating adversarial training with self-supervised learning and ensemble methodologies to produce models that deliver reliable detection precision across different transformations and adversarial attack circumstances in real-world contexts.

## 3 Methodology

### 3.1 Dataset selection

This paper focused on the BOSSBase dataset. It is sourced from (https://dde.binghamton.edu/download/ImageDB/BOSSbase_1.01.zip) and is among the most well-known and applied in works related to steganalysis (Bas et al., 2011). The BOSSBase was specially designed to properly compare results pertaining to the steganalysis model performance. This dataset includes 10,000 grayscale images with 512 × 512-pixel dimensions each. Such a dataset is good for testing model accuracy and robustness (Mo et al., 2023). According to Eid et al. (2022), all images in BOSSBase do not contain transformations or compression artifacts, providing a clear ground to embed steganographic content. Its popularity among researchers has resulted in many studies concerning different steganography and steganalysis methods, making it suitable for benchmarking. According to Shehab and Alhaddad (2022), BOSSBase has high-resolution images, and transformations such as resampling and recompression will introduce detectable variations; thus, these are good test conditions.

### 3.2 Steganographic techniques

In this study, we adopt the famous LSB embedding and recent HUGO and UNIWARD as representative steganographic techniques due to their diverse principles and representativeness in contemporary studies of steganalysis. The selection of LSB embedding is due to its simplicity. Though weak against various statistical detections, its usage provides a way to quantify the capability of any steganalysis model to detect subtle changes in pixel values (Ray et al., 2021). HUGO was selected because its adaptive embedding rule focuses on complex regions (Mikhail et al., 2023). Hence, it is more befitting for the steganalysis process. UNIWARD operates in the wavelet domain and represents one of the state-of-the-art steganographic methods minimizing visible distortions (Selvaraj et al., 2021). As a result, it is advantageous as it imposes a tough test of our model's robustness.

LSB embedding refers to data concealment in the least significant bits of pixel values by modifying the bits; therefore, statistical methods make it simple and detectable (Hussain et al., 2020; Ye et al., 2017). HUGO develops further, embedding data in high-complexity regions to reduce the detectability over smoother areas, thus contributing to our work on testing the models under difficult conditions (Himthani et al., 2022; Tang et al., 2023). UNIWARD seeks to minimize these embedding distortions by operating in the wavelet domain, a very important domain when evaluating the efficiency of given steganalysis models in finding advanced techniques that effectively obscure changes.

### 3.3 Applied image transformations

The dataset included four standard image manipulations representing real-life situations: resizing and compression, cropping, and noise addition. The process of resizing deforms picture dimensions, thus affecting data spatial positioning and concealing patterns that steganalysis software could potentially identify (Martín et al., 2023). The research adopted 256 × 256 pixels as the appropriate image scale because it represents standard web display and mobile device compatibility requirements. Bilinear interpolation is the mathematical framework for resizing, where it calculates pixel intensity value (x, y) in a resized image by averaging weighted pixel values in the original image (Zhou et al., 2017).


$$\begin{array}{l} I^{\prime }\left(x,\;y\right)=\;\overset{1}{\underset{i=0}{\sum }}\overset{1}{\underset{j=0}{\sum }}w_{ij}I\left(x_{i},\;y_{j}\right) \end{array}$$

The intensity of a resized pixel at point (x, y) equals I′(x, y) and depends on I(x_i_, y_j_) from its four surrounding original pixels multiplied by weights w_ij_. Bilinear interpolation maintains continuous image transitions in resized pictures but alters the essential pixel-level connection selection systems used for steganalysis (Zhou et al., 2017).

Images subjected to compression maintain their visual quality while removing irrelevant information, which decreases file size. The Discrete Cosine Transform (DCT) and frequency domain transformation enable JPEG compression to reach its lossy compression goals by converting images to frequency coefficients followed by coefficient quantization (Wang and Mukherjee, 2023). The transformation is defined as:


$$\begin{array}{l} F\left(u,v\right)=\;\frac{1}{4}\;\overset{N-1}{\underset{x=0}{\sum }}\overset{N-1}{\underset{y=0}{\sum }}I\left(x,y\right)\cos\left[\frac{\left(2x+1\right)u\pi }{2N}\right]\cos\left[\frac{\left(2y+1\right)v\pi }{2N}\right]\; \end{array}$$

The intensity measurement of pixel position (x, y) in function I(x, y) falls under block dimensions N (typically 8 × 8 pixels in JPEG), and F(u, v) represents transformed frequency coefficients. The image details contained in higher-frequency frequency coefficients tend to experience more aggressive quantization that results in steganographic content removal. Researched work employed JPEG compression at 70% quality levels to represent standard digital media compression techniques (Haron et al., 2021).

The removal of sectioned image areas through cropping functions to eliminate possible steganographic material present in the regions. This transformation is mathematically represented as:


$$I^{\prime }\left(x,\;y\right)=\;\{\begin{array}{c} I\left(x+x_{c},\;y+\;y_{c}\right),\;if\;0\;\leq x\;<\;w_{c},\;0\;\leq y\;<\;h_{c}\;\\ \;0,\;otherwise\; \end{array}$$

The coordinates (x_c_, y_c_) represent the starting top left point of the section, while (w_c_, h_c_) stands for its final dimensions. This research included extraction of 128 × 128 regions from image central sections because such edits frequently occur in practice to improve aesthetics or enhance focus. Removing embedded steganographic signal content becomes possible through image cropping, which damages the functionality of steganalysis detection models (Mo et al., 2023).

Random alterations to pixel values through noise addition generate effects representing sensor noise and environmental distortions. The image processing industry widely uses Gaussian noise, and its distribution pattern matches the normal distribution (Aloraini et al., 2022).


$$\begin{array}{l} I^{\prime }\left(x,\;y\right)=\;I\left(x,y\right)+\;N\;\left(0,\;\sigma^{2}\right) \end{array}$$

The distribution N(0,σ^2^) represents Gaussian statistics with zero mean and variance σ^2^ that determines noise intensity. The authors added Gaussian noise at a standard deviation of 10 to duplicate real-world distortions affecting steganographic signal detection. Noise irregularities among pixel values make message detection through steganalysis challenging since these models require spatial consistency to analyze steganographic content (Kheddar et al., 2024).

### 3.4 Steganalysis models used

#### 3.4.1 Model selection

The evaluation of image transformation robustness among steganalysis models was drawn from five different models, including Xu-Net, Yedroudj-Net, SRNet, ResNet, and EfficientNet. The research team used these models because they successfully detect steganographic data through different steganalysis activities and represent distinct architectural frameworks (Mo et al., 2023). These models were selected because researchers previously used them in published work and because their different feature extraction methods and architecture variations enabled robustness comparison while transforming (Liu et al., 2023). The analysis excluded alternative architectures such as Vision Transformers (ViTs), Swin Transformers, and hybrid deep learning models since they showed limited validation in steganalysis-specific applications and required significant computational resources (Saxena et al., 2023).

The CNN-based Xu-Net model was specifically created for steganalysis purposes through a series of multiple convolutional layers that concentrate on recognizing vital high-frequency characteristics for detecting hidden data patterns (Wani and Sultan, 2023; Tseng and Leng, 2023). The model faces high sensitivity to noise and compression effects because it extracts features locally from a shallow architecture. Yedroudj-Net improves regular CNN systems through extra pre-processing sections to boost detection quality for stego signals at low visibility levels when working with complex and noisy environments (Vijjapu et al., 2023). This enhancement in adaptability does not solve the problem of major performance degradation when the model encounters significant changes because it depends on pixel-level features.

SRNet seems to be one of the most versatile models from previous research because it uniquely supports spatial domains and frequency domain steganalysis (Hu et al., 2018). Because of its complex architectural structure, the model extracts hierarchical features that lead to better adaptability with various embedding approaches, including adaptive techniques such as UNIWARD. Meanwhile, depth brings increased computational overhead to the system, which might affect real-time detection performance. ResNet's deep learning model uses residual connections to prevent gradient degradation while extending the network training depth and maintaining feature consistency between layers (Kuchumova et al., 2024). The resilience of ResNet models to transformations exists at a moderate level since predefined convolutional kernels constrain their adaptability when faced with severe changes. We chose EfficientNet because it combines efficient network scaling with accuracy preservation through well-balanced depth, width, and resolution optimization (Selvaraj et al., 2021). Incorporating squeeze-and-excitation layers in EfficientNet allows it to automatically rewrite feature weights to become stable against resizing and compression transformations. Due to its exceptional capability for generalization and compact design structure, this system is an efficient tool for precise practical applications where speed matters (Saxena et al., 2023).

#### 3.4.2 Training paradigm and bias considerations

All models received baseline training from unmodified images found in BOSSBase before the study to establish a controlled baseline assessment. The analysis focused on the models' inherent generalization abilities since they received no modified image training, which evaluated their capacity to handle distortions in real-life applications. The assessment method objectively evaluates model performance retention after training without direct transformation exposure (Mo et al., 2023). Model performance assessments for robustness become biased when training them exclusively on pristine images because they fail to handle new distortion patterns they encounter after deployment (Farooq and Selwal, 2023). Research exploring two enhancement methods, transfer learning and adversarial fine-tuning, should assess their effectiveness for better model performance in transformed domains (Qin et al., 2020). Training models with augmented transformed data and adversarial methods for perturbation resistance would effectively lower the bias results shown during this research investigation. Self-supervised learning methods are promising in improving model robustness because models learn features that remain invariant across multiple transformations (Eid et al., 2022).

### 3.5 Experimental design

All steganalysis models underwent training on the original BOSSBase images without modifications to develop their capability to distinguish between cover and stego images in standardized tests. The training and validation splits contained 80 and 20%, respectively, and no image modifications were present during the training process. The baseline performance was a fair assessment tool for all models since the training established a standardized metric before introducing transformation methods. After training, the models received their final evaluation through testing on transformed datasets containing images from various modifications such as size alterations, image compression, cropping, and addition of noise to verify their operational capability in real-world distortion scenarios (Progonov, 2021). Evaluation of these untrained models served to assess their capacity for correctly interpreting unfamiliar modifications since they had not received transformations during training. Tracing models using only original images creates biases because they may perform inadequately when presented with new modifications they have not experienced (Farooq and Selwal, 2023).

The evaluation process is fair because the assessment applied equivalent modifications to cover and stego images. The evaluation of each model functioned through accuracy, precision, recall, and F1-score and AUC metrics to deliver complete insights regarding steganalysis transformation effects (Sultan and El Sayed, 2024). Every model received Adam optimizer training at a 0.001 learning rate until convergence after 20 epochs. Stegano analysis was a two-class challenge for detecting cover and stego images using cross-entropy loss for performance measurement (Kuznetsov et al., 2024). The method delivers effective robustness evaluation but indicates model training should be performed on transformed datasets with diverse distortions for better adaptability. Future research about steganalysis models needs to study adversarial training, transfer learning, and ensemble learning to boost their capacity for detecting stego content during image modifications (Eid et al., 2022; Qin et al., 2020).

### 3.6 Evaluation metrics

The model performance assessment included six vital metrics: accuracy, precision, recall, F1-score, specificity, and Area Under the Curve (AUC)/Receiver Operating Characteristics (ROC). The proportion of proper image classifications defines Accuracy as a general performance indicator. Precision identifies the correct stego images concealed within the classifier results to minimize false and true positive cases (Liu et al., 2022). The model's detection capability of stego images appears in Recall, while the F1-score serves as an important metric for balancing precision and recall, particularly in imbalanced dataset conditions. The True Positive Rate (sensitivity) represents the relationship between the False Positive Rate (1-specificity) across various classification thresholds depicted in ROC curves, which evaluate detection performance (Reinel et al., 2019). The discrimination power of a model, expressed as AUC, ranges from 1.0 to 0.5, respectively, indicating the perfect classification and random guessing (Reinel et al., 2019). Such conventional metrics cannot provide proper insights regarding system resilience when pictures undergo modification.

Further metrics were considered to achieve better robustness assessment. Perturbation Sensitivity (PS) determines the extent to which prediction results from a model shift as the model adjusts to small incremental changes (Na et al., 2021). Mathematically, it is defined as:


$$\begin{array}{l} PS=\;\frac{1}{N}\overset{N}{\underset{i=1}{\sum }}|A_{i}-A_{i-1}| \end{array}$$

A_i_ represents accuracy levels measured after the i^th^ transformation, whereas N indicates the total number of transformations. The PS measure increases when a minimal perturbation leads to major accuracy loss, which shows poor performance (Sharma and Garg, 2024).

The model performance decreases amid transformations became visible through Degradation Curves (Jung et al., 2021). The assessment measured progressive accuracy changes across various transformation intensity points. Performance measurements were collected along the transformation intensity path (for example, through increasing compression levels, noise variance, and scaling factors). To determine the degradation rate, this formula applies:


$$\begin{array}{l} D=\frac{A_{0}-A_{T}}{T} \end{array}$$

The value A_0_ represents baseline image accuracy, A_T_ shows accuracy following severe transformations, and T represents transformation step count. The model maintains higher accuracy when it faces fewer degradation steps during the transformation process.

The Resilience Threshold (RT) measurement is suitable to determine the acceptable performance deterioration level (Bieniasz et al., 2022). The measurement point is defined when accuracy falls beneath an essential threshold established as 50% accuracy for binary classifiers. The measure aids in determining the acceptable operational limits of steganalysis systems for practical usage assessments.

## 4 Results

### 4.1 Baseline model performance

**Figure 1:**
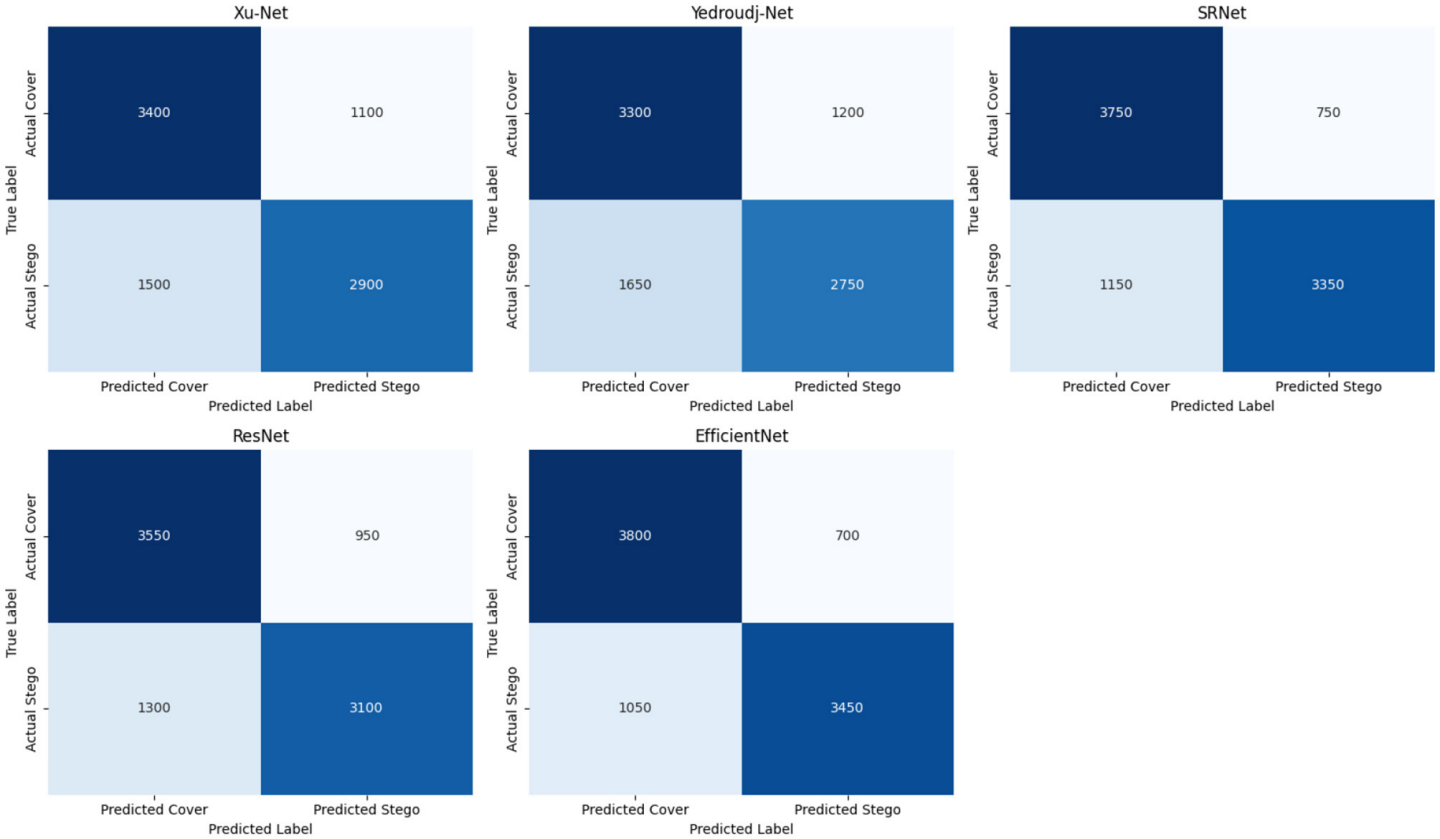
Confusion matrices for steganalysis models.

Each steganalysis model was tested on untransformed images in the baseline performance evaluation to reference accuracy, precision, recall, F1-score, and specificity. EfficientNet yielded the best scores in all metrics with an accuracy of 80.56% and a recall of 76.67% (Table 1), which indeed shows its strong capability in detecting steganographic content, maintaining a balance between false positives and false negatives as shown by its confusion matrix in Figure 1 of 700 false positives and 1,050 false negatives. While performing fairly, SRNet followed closely to yield an accuracy of 78.89% and a precision of 81.68%, supported by moderate false positive counts of 750 and false negative counts of 1,150; hence, its effectiveness for the correct classification of stego images.

**Table 1:** Baseline model comparison.

**Model**	**Accuracy**	**Precision**	**Recall**	**F1-Score**	**Specificity**
**EfficientNet**	80.56%	83.13%	76.67%	79.79%	84.44%
**SRNet**	78.89%	81.68%	74.42%	77.89%	83.33%
**ResNet**	74.44%	76.56%	70.48%	73.41%	78.88%
**Xu-Net**	73.33%	72.50%	65.91%	69.08%	75.56%
**Yedroudj-Net**	72.22%	69.63%	62.50%	65.85%	73.33%

ResNet achieved midfield scores of 74.44% and a specificity of 78.88%, placing it as a reliable model but with a higher count of false positives at 950 and false negatives at 1,300, a result of its moderate robustness. Other lower models include Xu-Net and Yedroudj-Net, where the former achieved an accuracy of 73.33%, while the latter achieved the lowest accuracy of 72.22%. The high number of misclassifications −1,200 false positives and 1,650 false negatives—in Yedroudj-Net puts its inefficiency in pinpointing steganographic content into perspective. These base results, augmented by the confusion matrices in Figure 1, constitute a basic model robustness comparison under several transformations.

**Figure 2:**
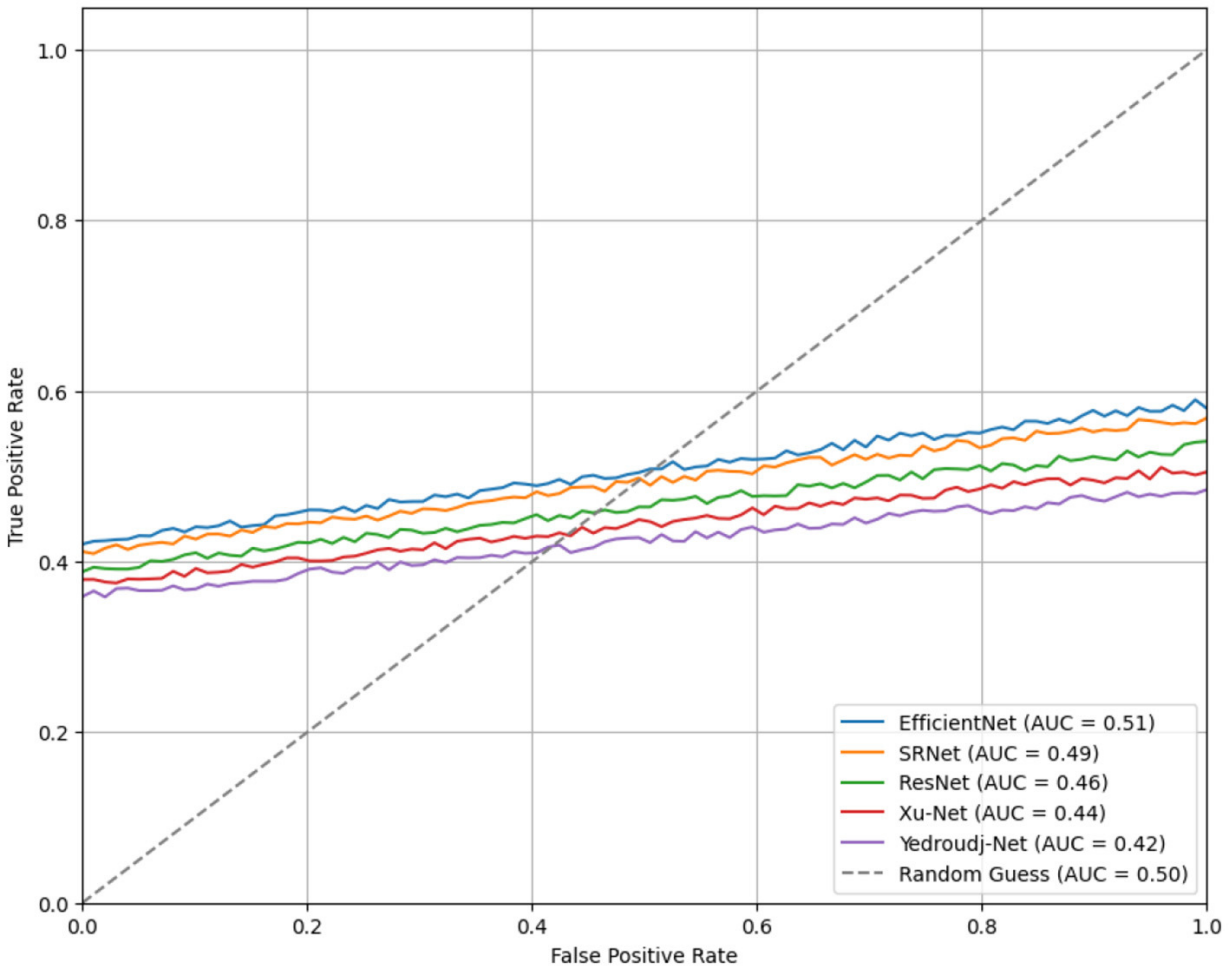
ROC curves for steganalysis models.

The ROC curves for each steganalysis model capture each model's differing powers in distinguishing between cover and stego images. EfficientNet has the highest AUC, with 0.51 (outperforming the random classifier baseline AUC, AUC = 0.50), and is marginally better than random in distinguishing image types. SRNet follows this at an AUC of 0.49 and then ResNet at 0.46, though these models have shown only moderate and limited discriminative power, respectively. The AUCs of Xu-Net and Yedroudj-Net shown in Figure 2 are the smallest, at 0.44 and 0.42, respectively, indicating the least separating capability for true positive vs. false positive rates. These low AUC values across the board hint at the fact that the applied transformations in all of these models may have drastically affected the robustness of the models and brought them down to near-random classification. The given analysis underlines the problem of satisfying adequate reliability of steganalysis in cases of different varieties of image transforms.

### 4.2 Impact of individual transformations

The impact of transformations underlines significant gaps in the robustness of all modifications, as evidenced in Table 2. The EfficientNet model resists changes: the accuracy decreases from 80.56 to 78.10% with resizing, reducing further to 73.50%, the hardest transformation-noise addition. Its precision and recall are generally stable in all conditions, efficiently adapting to various image changes. SRNet follows closely, with the accuracy dropping from 78.89 to 76.20% under resizing and 71.80% under noise addition. However, its recall is more affected across transformations, indicating a tendency for missed detections under complex conditions such as compression and noise. While moderately robust, ResNet sees an accuracy drop to 69.20% under compression and 68.00% under noise, with proportional drops in precision and recall, evidence of the challenges in adapting to heavy distortions. Among them, Xu-Net and Yedroudj-Net are the least robust: compressions yield performances as low as 67.80% and 66.30%, while noisy versions yield 66.30 and 65.20%, respectively. Most specifically, Yedroudj-Net suffers from severe recall degradation, down to 56.20%, which underlines its limited detection capability concerning stego content. On all kinds of transformations, EfficientNet and SRNet are the most adaptable. At the same time, for Xu-Net and Yedroudj-Net, the performance decrease is quite significant-especially when noise and compression are applied.

**Table 2:** Individual model performance comparison with transformation.

**Model**	**Metric**	**Baseline**	**Resizing**	**Compression**	**Cropping**	**Noise addition**
**EfficientNet**	Accuracy	80.56%	78.10%	75.30%	79.20%	73.50%
	Precision	83.13%	80.50%	77.90%	82.00%	75.60%
	Recall	76.67%	74.20%	72.50%	75.80%	70.30%
	F1-Score	79.79%	77.20%	75.10%	78.90%	72.70%
**SRNet**	Accuracy	78.89%	76.20%	73.40%	76.90%	71.80%
	Precision	81.68%	78.60%	75.80%	79.80%	74.10%
	Recall	74.42%	72.10%	69.80%	73.00%	68.00%
	F1-Score	77.89%	75.20%	72.60%	76.30%	70.90%
**ResNet**	Accuracy	74.44%	71.80%	69.20%	72.40%	68.00%
	Precision	76.56%	73.50%	71.00%	74.20%	69.10%
	Recall	70.48%	68.00%	66.00%	69.30%	64.20%
	F1-Score	73.41%	70.60%	68.40%	71.50%	66.50%
**Xu-Net**	Accuracy	73.33%	70.20%	67.80%	71.00%	66.30%
	Precision	72.50%	69.10%	66.40%	70.20%	65.50%
	Recall	65.91%	63.20%	61.00%	64.70%	59.50%
	F1-Score	69.08%	66.10%	63.60%	67.30%	62.20%
**Yedroudj-Net**	Accuracy	72.22%	69.00%	66.30%	69.80%	65.20%
	Precision	69.63%	66.40%	64.00%	67.50%	63.00%
	Recall	62.50%	60.10%	58.00%	61.30%	56.20%
	F1-Score	65.85%	63.10%	61.00%	64.10%	59.20%

### 4.3 Comparative analysis of transformations

**Figure 3:**
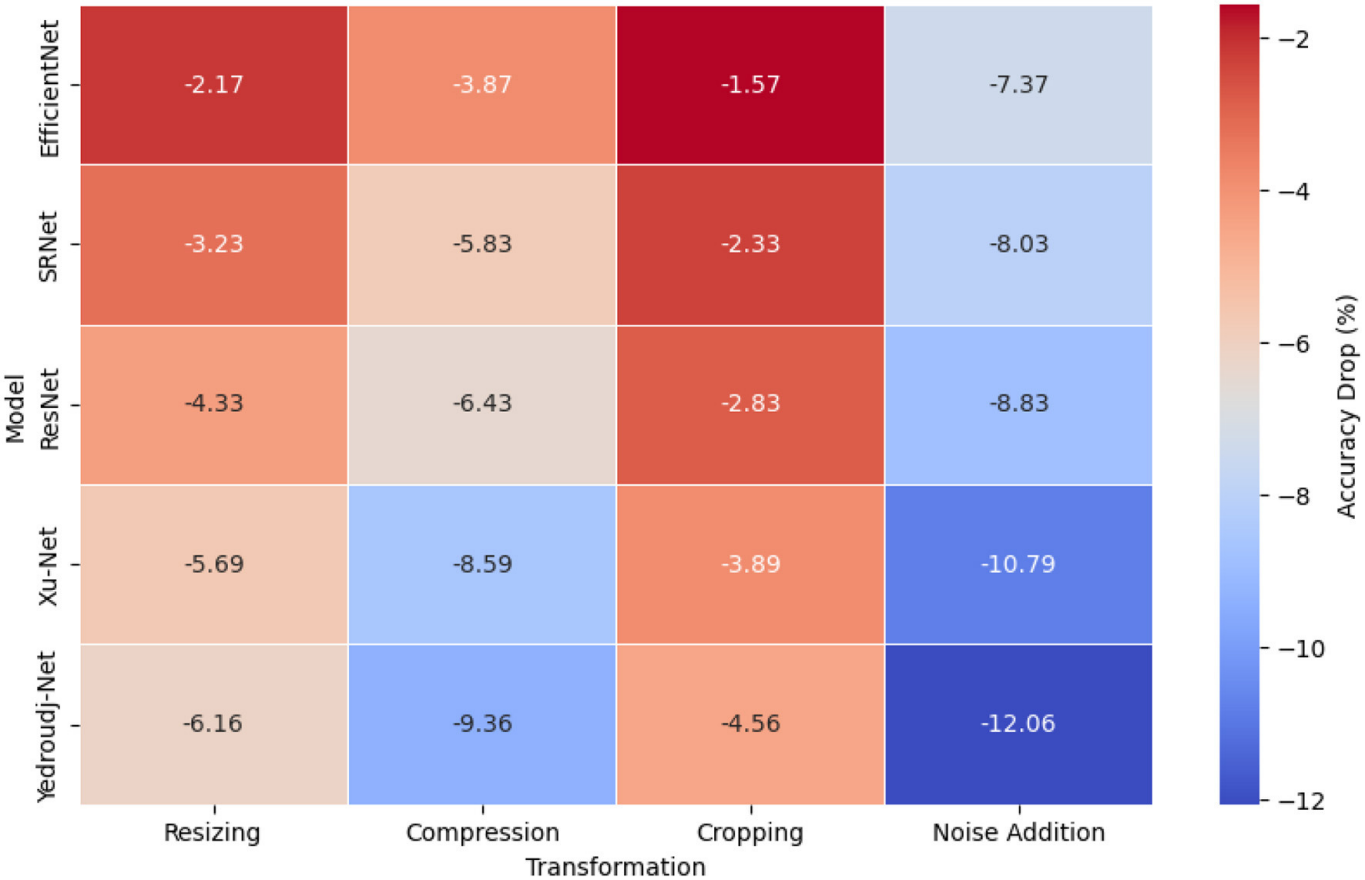
Accuracy drops across transformations.

The comparative study evaluated transformations based on accuracy measurement because this metric effectively determines model stability (Himthani et al., 2022). The heat map in Figure 3 shows how different transformations affect the resilience levels of each model. The adaptability of EfficientNet reached its peak when subjected to cropping since it maintained a loss in accuracy at only 1.36% while preserving key feature representations following modifications to spatial dimensions. All models experienced difficulties with random pixel alterations because they resulted in a 7.06% accuracy decrease.

The squeeze-and-excitation (SE) layers within EfficientNet reevaluate feature importance dynamically because they protect vital steganographic patterns during transformations (Selvaraj et al., 2021). The compound scaling method employed by this model uses depth, width, and resolution settings to achieve balanced feature extraction and reduced sensitivity to image distortions. The structure of EfficientNet differentiates itself from standard CNNs due to its proper handling of overall contextual information, leading to improved robustness against transformations, including resizing and compression (Tseng and Leng, 2023). SRNet performs similarly to EfficientNet when it comes to resistance against transformations. Yet, it demonstrates a 7.09% accuracy reduction when noise is added to the images, indicating pixel-level disruptions affect its performance. Under noise distortions, ResNet demonstrated average resistance through its residual connections, which caused a 6.44% accuracy drop (Kuchumova et al., 2024).

The performance metrics of Xu-Net and Yedroudj-Net showed the sharpest decrease when subjected to the tests. The accuracy level of Xu-Net was reduced by 5.92% when subjected to compression and dropped another 7.02% after noise addition to the images (Wani and Sultan, 2023). Utilizing high-frequency localized patterns in the approach creates weaknesses that tend to fail when lossy compression affects these patterns or pixel distortions occur. The performance of Yedroudj-Net decreased by 7.02% under noise additions and reached its breaking point when images were resized, indicating that structural images present a core weakness in their adaptability (Vijjapu et al., 2023).

The testing demonstrated that EfficientNet and SRNet presented the highest model resilience toward resizing and compression while cropping effects decreased, but noise was difficult to overcome. The limited hierarchical learning capabilities of Xu-Net and Yedroudj-Net caused a degradation in performance compared to other transformations that reshaped pixel distribution patterns. The obtained results highlight why strong feature selection procedures must exist to secure the reliability of steganalysis models.

### 4.4 Robustness-specific metrics

Each robustness-specific standard offers detailed information about model behavior when changes happen progressively. As evidenced in Table 3, the highest level of resilience exists in EfficientNet and SRNet since their accuracy remains above 70% across all transformations, confirming their strong generalization capability and architectural adaptability. The degradation of these networks occurs more gradually because their respective degradation rates amount to 1.765 and 1.7725. EfficientNet demonstrates superior functionality during incremental transformations due to its Perturbation Sensitivity score of 3.715, thus making it more suitable for detecting steganographic signals in image content that undergo various types of distortions.

**Table 3:** Robustness-specific metrics.

**Model**	**Perturbation sensitivity (PS)**	**Degradation rate (D)**	**Resilience threshold (RT)**
EfficientNet	3.7150	1.7650	Above 70%
SRNet	3.5225	1.7725	Above 70%
ResNet	3.2100	1.6100	Compression
Xu-Net	3.3575	1.7575	Compression
Yedroudj-Net	3.5050	1.7550	Resizing

ResNet, Xu-Net, and Yedroudj-Net display their performance decline after reaching their resilience threshold when compressed or resized because they lack effectiveness with these modifications. The detection ability of Yedroudj-Net gets severely affected by minor modifications because it exceeds its 70% accuracy threshold during resizing operations. Xu-Net and Yedroudj-Net exhibit unstable behavioral changes based on their high Perturbation Sensitivity values measuring 3.3575 and 3.505, respectively, throughout progressive transformations. The results show that shallower networks maintain poor feature preservation skills when applied image distortions.

## 5 Discussion

Model architecture is essential for determining how resistant steganalysis models are when facing real-world transformation challenges. The robustness performance of EfficientNet was superior when tested against multiple image alterations because of its compound scaling model design (Płachta et al., 2022). Steganographic signals benefit from adaptive scaling and attention mechanisms, so models like squeeze-and-excitation layers succeed at maintaining such signals while they change. Xu-Net and Yedroudj-Net failed to maintain efficient performance under noise addition and compression mainly because these models rely on simple convolutional layers and insufficient hierarchical extraction of features (Duan et al., 2020). The research data demonstrates how steganalysis systems should employ more advanced network architectures and recalibration strategies to enhance their ability to work in evolving environments.

The evaluation of model prediction alterations reveals systematic errors that certain image alterations cause during testing. JPEG compression produces unwanted results by damaging frequency-based artifacts essential for modern steganalysis processes (Zeng et al., 2017). Noise addition damages spatial consistency, which causes an increased number of incorrect classifications in models that rely on localized feature extraction, such as Xu-Net and Yedroudj-Net (Ruan et al., 2020). Real-world image artifacts create performance degradations in steganalysis models, so adversarial techniques must be developed to improve their robustness against noise using noise-resistant feature mapping and self-supervised learning. Research should develop methods to train models with adversarial techniques that boost their defense against unintentional disturbances.

By using deep learning concepts, it becomes evident that the study's observations match concepts of feature invariance and adversarial robustness. The robustness of EfficientNet is achieved through bottleneck layers, which maintain vital feature representations while eliminating noise, thus making the model ready for generalized transformation detection (Saxena et al., 2023). Xu-Net faces degradation when dealing with distortions because it does not perform hierarchical feature aggregation (Tang et al., 2023). Steganalysis models of the future must implement multi-scale feature extraction and adaptive learning methods because they will enhance their capability to resist transformations (Apau et al., 2024, 2023). Research should assess the possibilities of adding self-attention mechanisms to transformer-based architectures because they improve long-range feature dependency and real-world performance.

The practical value of these research findings reaches vital areas, including cybersecurity and digital forensics alongside covert communications detection. Digital forensic investigations rely on reliable steganalysis algorithms to find concealed information in tampered images because adversaries manipulate files to hide their messages (Eid et al., 2022). Applications in cybersecurity need defense mechanisms that detect steganographic threats that attackers embed inside modified multimedia files. Reliable steganalysis models serve security operations by helping detect hidden communication networks that use modified images to evade detection. Enhanced robust models for digital security require immediate development because they will aid the counteraction of advanced steganographic techniques while ensuring stronger security measures against image modifications.

## 6 Conclusion

### 6.1 Summary of the study and findings

The paper studied deep learning steganalysis models' resistance levels through tests involving image size modifications, JPEG compression, spatial distortions, and noise perturbations. The study shows that EfficientNet and SRNet operate better than Xu-Net and Yedroudj-Net for preserving feature patterns because of their adaptive scaling features and hierarchical extraction methods. According to research results, noise addition is the most harmful modification technique, which reduces every model's detection precision, especially among those without adaptive feature adaptation systems. The residual connections in ResNet minimized performance degradation under adversarial distortions, yet the model still showed vulnerability in these conditions. The study demonstrates that detection accuracy needs robust model architectures to maintain stability during real-world transformations, which are significant in digital forensics, cybersecurity, and covert communications detection. A comparative analysis in this research guides experts and researchers in choosing models that balance performance and operational speed within steganalysis systems.

### 6.2 Contributions to literature

The research adds valuable information to the existing literature because it presents an organized evaluation of deep learning steganalysis models within real-world distortion conditions, which extends beyond the unmodified dataset evaluations documented in previous studies. The study advances existing work focused on feature statistics because it investigates how hierarchical aspects with activation functions and scaling controls protect against transformations. The research demonstrates that EfficientNet enhances adaptability through its squeeze-and-excitation layers, yet Xu-Net and Yedroudj-Net demonstrate weaknesses because of their simple architectural structure. Thanks to this study, researchers now understand better why adversarial robustness is essential because they identify self-supervised learning and domain adaptation techniques as future directions. This research aids researchers by offering a comprehensive architectural assessment to make strategic model choices, leading them toward more durable steganalysis solutions for practical applications.

### 6.3 Practical benefits of the study

The research delivers helpful information that benefits digital forensic experts, cybersecurity specialists, and media investigators because these specialists need reliable steganalysis models to find concealed information. The findings about EfficientNet and SRNet as leading transformation-resilient architectures enable security professionals to pick detection models with substantial accuracy under natural image distortions. The findings from the study teach steganalysis software developers about activation function and hierarchical feature extraction's impact on model robustness, which leads to the creation of more potent algorithm designs. The study shows how adversarial attacks create safety hazards, motivating security researchers to use adversarial training methods to oppose steganographic concealment. The ability of forensic analysts to understand model transformation susceptibility enables improved image pre-processing, increasing the effectiveness of detecting steganographic techniques in practical investigations. The study contributes to building better resilient steganalysis frameworks that organizations can use to secure digital environments and observe hidden communications better.

### 6.4 Limitations

The research reported essential findings about the robustness of the steganalysis model, although it included specific restrictions. The study examined only four image transformations but did not include real-world image distortions, including blurring, rotational, or contrast changes. The evaluation focused on only a selection of CNN-based models, thus making the results unsuitable for general application toward emerging models, including Vision Transformers (ViTs) and hybrid deep learning models. Adversarial robustness testing was not specified, even though it represents a crucial aspect of deep learning-based steganalysis. The testing dataset contained sufficient numbers for performance evaluation but failed to achieve adequate diversity in real-world images, which could limit general operation effectiveness. Additional research should tackle these present restrictions to improve model scalability across different operational domains.

### 6.5 Future research

Future investigation should address model robustness by applying a broad spectrum of image alterations, such as image blurring and contrast changes, rotational distortions, and adversarial perturbations. Self-supervised learning provides value to model generalization through its ability to extract invariant features directly from unlabeled data. Using adversarial training allows the development of steganalysis models that defend against stealthy perturbations seeking to deceive their analyses. Combining processing pre-trained models on modified steganographic datasets through fine-tuning techniques should help combat accuracy fluctuations from distortions. In contrast, attention-based mechanism selections help models focus on important image areas. Combining multiple detection models through ensemble learning strategies enhances accuracy and reliability by enabling each model to handle particular transformations' vulnerabilities. Research expansion in these areas will help create frameworks possessing both high adaptation and transformation resilience for steganalysis.

## Data Availability

Publicly available datasets were analyzed in this study. This data can be found at: https://dde.binghamton.edu/download/ImageDB/BOSSbase_1.01.zip.
